# Vaccinating in Different Settings: Best Practices from Italian Regions

**DOI:** 10.3390/vaccines13010016

**Published:** 2024-12-28

**Authors:** Carlo Signorelli, Flavia Pennisi, Anna Carole D’Amelio, Michele Conversano, Sandro Cinquetti, Lorenzo Blandi, Giovanni Rezza

**Affiliations:** 1 Faculty of Medicine, University Vita-Salute San Raffaele, 20132 Milan, Italy; signorelli.carlo@hsr.it (C.S.); a.damelio@studenti.unisr.it (A.C.D.); rezza.giovanni@hsr.it (G.R.); 2 National Immunization Technical Advisory Group (NITAG), Atlanta, GA 30033, USA; 3 PhD National Programme in One Health Approaches to Infectious Diseases and Life Science Research, Department of Public Health, Experimental and Forensic Medicine, University of Pavia, 27100 Pavia, Italy; 4 Prevention Department, Local Health Authority of Taranto, 74123 Taranto, Italy; michele.conversano@asl.taranto.it; 5 Prevention Department, Local Health Authority “ULSS 1 Dolomiti”, 32100 Belluno, Italy; sandro.cinquetti@aulss1.veneto.it; 6 Health Direction, Istituti Ospedalieri Bergamaschi, 24046 Bergamo, Italy

**Keywords:** vaccination, providers, Italy, national vaccination plan, immunization schedule

## Abstract

Background: The success of vaccination programs depends on a complex interplay of logistical, social, and structural factors. The objective of this study was to analyze the different approaches to vaccine administration implemented by several Italian regions since the onset of the SARS-CoV-2 pandemic. Methods: After careful qualitative review of information gathered from scientific articles, official reports (grey literature), contact with regional health authorities, and local health departments, five vaccination strategies across several Italian regions focusing on alternative vaccine providers and/or settings were identified. The innovative practices implemented by different actors covered specific topics and were then examined and described in detail. Results: In Veneto, where prevention departments were the main actor, herpes zoster vaccination coverage for the 65-year-old cohort increased from 44.4% to 54.9%; in Tuscany, family pediatricians administered 64% of all childhood vaccines; in Liguria, pharmacies delivered 70.1% of COVID-19 vaccines, while vaccinating in schools in Taranto led to higher human papilloma virus vaccination rates compared to regional and national averages. Finally, in all the regions, hospitals focused on vaccinating healthcare workers and vulnerable populations. Conclusions: The positive outcomes of these five experiences may, in a context of limited resources, encourage other national and international entities to adopt innovative practices, which offer new perspectives beyond the traditional delivery methods (i.e., local health authority vaccination centers for childhood and adolescent immunizations, and family doctors for adults and the elderly). These strategies suggest the efficacy of specific local approaches favored by regional autonomy in optimizing vaccine distribution and coverage.

## 1. Introduction

The success of vaccination campaigns, particularly in achieving high coverage rates, depends on a complex interplay of logistical, social, and structural factors. Vaccine availability remains a cornerstone of successful campaigns [1], but the challenges extend beyond the supply chain. The costs associated with vaccine procurement and distribution, the capacity of the healthcare workforce, and the level of collaboration between governmental bodies, healthcare providers, and other stakeholders all play a critical role [2,3]. In addition, public vaccine hesitancy due to factors such as misinformation, mistrust of medical authorities, and cultural beliefs can significantly hinder the achievement of immunization goals [4,5,6]. Moreover, the presence of effective and well-distributed vaccination providers is essential to ensure that vaccines are accessible to all sectors of the population, particularly in underserved or rural areas [7].

In Italy, the vaccination system is governed by the National Vaccination Prevention Plan (PNPV) [8], which outlines strategies to prevent infectious diseases through immunization. Vaccination requirements are tailored to specific age groups and at-risk populations, ensuring widespread protection and adherence to national public health goals. For children and adolescents, vaccination is mandatory as per Law 119/2017 [9], which includes immunization against diseases such as diphtheria, tetanus, pertussis, poliomyelitis, hepatitis B, Haemophilus influenzae type B, measles, mumps, rubella, and varicella (for those born after 2017) [10,11]. These vaccinations are offered free of charge as part of the Essential Care Levels (LEA—Livelli Essenziali di Assistenza) [12], ensuring equitable access for all. Compliance is essential for school enrollment, with exception allowed only for valid medical reasons.

Certain vaccines are strongly recommended at the national level, though they are not legally mandatory. These include RSV prophylaxis for all newborns, HPV vaccines, flu vaccines, herpes zoster and pneumococcal vaccines for adults, and COVID-19 vaccines.

In addition to mandatory vaccinations, the National Vaccination Prevention Plan (PNPV) and the annually renewable Vaccination Calendar (Calendario vaccinale) [13] recommend certain vaccines for specific population groups [14], which regions can choose to expand or make more accessible [15]. Regions can offer recommended vaccines free of charge to additional groups or for broader coverage or in response to public health emergencies or specific epidemiological needs.

For example, in Apulia, with Regional Council Resolution (DGR) No. 241 of 18 February 2013 [16], the region implemented the active and free offering of the hepatitis A (HAV) vaccine. This measure, prompted by peaks in HAV infection recorded during 1996–1997 [17,18], made hepatitis A vaccination mandatory in Apulia and successfully reduced infection rates. For vaccines that are not mandatory or strongly recommended, citizens can decide based on medical advice or personal needs (e.g., travel requirements [19]). These include vaccines such as meningococcal B and ACWY for adolescents or travel vaccines (e.g., yellow fever, typhoid).

Italy’s decentralized health system provides a valuable context for examining the role of different vaccination providers. Each of its 19 regions and 2 autonomous provinces has developed unique approaches to immunization management [20]. These regional differences are influenced by several factors, including the administrative autonomy of regional governments, the establishment of agreements with healthcare partners—such as general practitioners (GPs), pharmacies, and nursing homes—and the availability of medical staff [21]. In addition, historical and cultural factors influence the adoption of certain vaccination strategies, with some regions relying on long-standing traditions of healthcare delivery.

The 2023–2025 PNPV [8,22] was introduced in August 2023 to maintain continuity with previous national plans [23], while incorporating the lessons learned from the large-scale COVID-19 vaccination campaign [24]. The COVID-19 pandemic presented relevant challenges but also provided valuable lessons in the management of large-scale vaccination efforts [25,26,27,28]. The PNPV encourages regions to expand their vaccination programs, using the infrastructure and methods developed during the pandemic to improve access and coverage. This plan aims to achieve equitable and efficient distribution of vaccines to different population groups, ensuring that all people have access to vaccines, regardless of their location or socio-economic status.

Despite the extensive literature on national vaccination programs, the impact of regional autonomy on immunization outcomes remains underexplored. This paper addresses this gap by analyzing how regional differences in governance, resource allocation and healthcare delivery influence vaccination strategies in Italy. By examining the innovative practices implemented by Italian regions, this work provides insights into how localized solutions can overcome barriers to immunization.

This paper analyzes five best practices from Italian regions that made significant contributions to the equitable, appropriate, and efficient delivery of vaccines to target populations. By examining these regional strategies, we aim to understand how certain regions have been more successful in overcoming challenges such as workforce shortages, vaccine hesitancy, and logistical constraints. In addition, this analysis highlights how innovative regional approaches, such as partnerships with non-traditional providers (e.g., pharmacies), have contributed to improving immunization coverage. Ultimately, the aim is to contribute to the wider discussion on how regional differences in healthcare management can influence the success of national immunization programs. Understanding these differences is crucial for designing policies that support more tailored and localized interventions, ultimately leading to better health outcomes at national and global levels.

Public health professionals, policymakers, and healthcare administrators involved in immunization programs at both national and regional levels are the target audience for this paper. Researchers and academics studying health systems, vaccine distribution, or public health inequalities may also find the insights valuable. Furthermore, this paper will benefit international organizations and stakeholders seeking to enhance vaccination coverage in complex or decentralized healthcare environments. By addressing both logistical and sociocultural challenges, the findings aim to guide the development of more equitable and effective vaccination policies.

## 2. Materials and Methods

### 2.1. Data Sources

Information concerning vaccination campaigns was primarily collected from the Department of Prevention of the Italian Ministry of Health and the National Immunization Technical Advisory Group (NITAG), which serve as pivotal observatories for the 19 regions and the 2 autonomous provinces [29]. The Ministry of Health publishes periodic national reports on vaccination coverage based on data submitted by regional health authorities and the National Vaccination Registry [30]. These reports provide vaccination coverage statistics and details on vaccination sites for all 19 Italian regions and the 2 autonomous provinces. Furthermore, some regions periodically publish their vaccination coverage data and, in some cases, details on vaccine administration sites. However, the consistency and standardization of this process is not uniform. For this study, supplementary data were collected from various sources, including scientific publications, grey literature [31], and reports produced by scientific associations. Moreover, direct requests for information were submitted by the NITAG presidency to relevant stakeholders. Many of these data, especially those obtained through direct requests, had not been previously published. Data validation was performed through triangulation methods, comparing information from multiple sources. Vaccination coverage rates from regional authorities were cross-checked with national data provided by the Ministry of Health’s National Vaccination Registry. Additional validation involved consultations with regional health officers and experts in immunization to ensure the accuracy of interpretations and conclusions.

### 2.2. Inclusion and Exclusion Criteria

The study focused on innovative vaccination strategies introduced at the regional or local level in Italy. Inclusion criteria were as follows:

1. Vaccination strategies that involved alternative providers or settings beyond traditional vaccination centers managed by local health authorities (e.g., pharmacies, schools, hospitals).
2. Programs implemented within the framework of the PNPV or responding to specific regional needs.
3. Availability of data to assess outcomes, such as vaccination coverage rates or population reach.

Exclusion criteria included the following:

1. Standard vaccination practices, such as routine childhood and adolescent immunizations administered by local health authority centers.
2. Vaccination strategies exclusively managed by general practitioners (GPs), as they are governed by a national agreement. The involvement of GPs is subject to a national agreement that is specific to that professional category [32].

### 2.3. Description of Innovative Strategies

**Figure 1:**
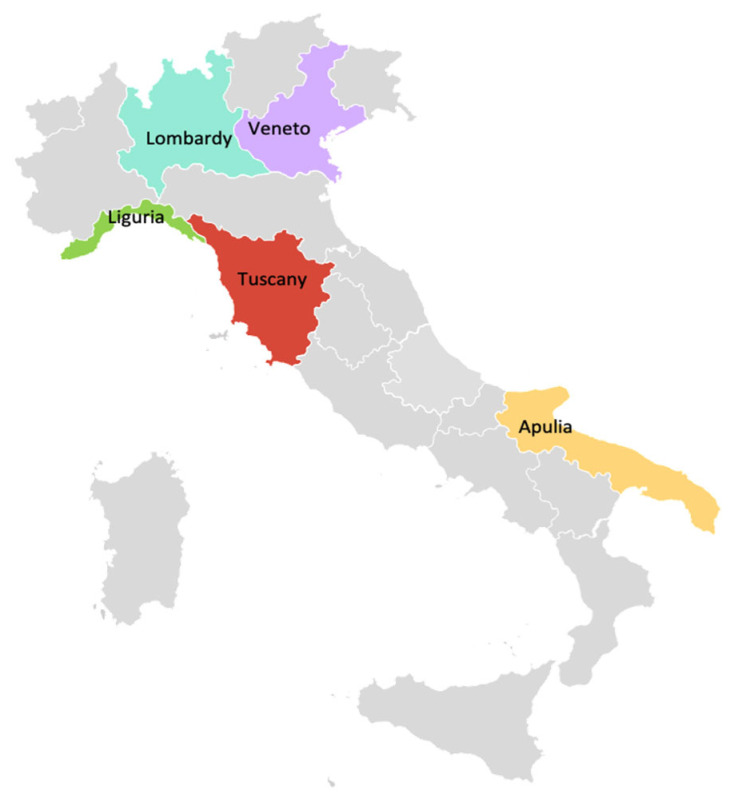
Map of Italy highlighting key regions discussed in the study: Lombardy, Veneto, Liguria, Tuscany, and Apulia.

Five innovative practices from 5 different regions (Figure 1) were selected based on their impact and feasibility:

1. Prevention departments in Veneto: Focused on increasing herpes zoster vaccination coverage through active recall strategies for the 65-year-old cohort.
2. Family pediatricians in Tuscany: Integrated pediatricians into the vaccination system, achieving high childhood vaccination rates.
3. Pharmacies in Liguria and Lombardy: Utilized pharmacies for influenza and COVID-19 vaccinations, supported by local agreements.
4. Schools in Taranto: Implemented HPV vaccination programs in educational settings to increase adolescent vaccination coverage.
5. Hospital settings nationwide: Targeted healthcare workers and vulnerable populations through hospital vaccination centers.

## 3. Results

Five paradigmatic best practices for vaccine administration introduced at the regional and local levels were identified, and the main results achieved are presented below.

### 3.1. Prevention Departments for Herpes Zoster Vaccination

**Figure 2:**
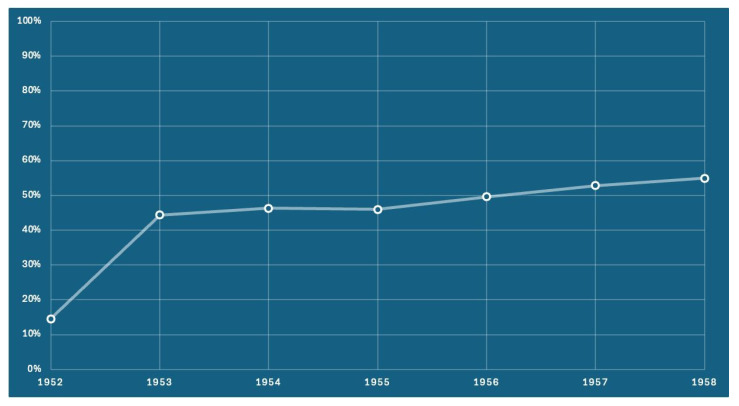
The adjusted vaccination coverage for the herpes zoster vaccine, presented by birth cohort. Coverage is calculated based on the receipt of either the attenuated vaccine or the first dose of the recombinant vaccine.

Vaccination centers, operating under the prevention departments of local health authorities, administer childhood and adolescent vaccines across all Italian regions. Despite the herpes zoster vaccination (HZV) being included in the national immunization schedule from as early as 2017, with initial coverage targets of 50% and expected incremental targets of up to 95%, national coverage rates remain below 15%. The Veneto region (northern Italy, with a population of 4.9 million inhabitants) has proactively addressed this issue in recent years by establishing a minimum coverage target of 50% for the 65-year-old cohort, as required by the General Directors of the Health Authorities [33]. Following the implementation of an active call strategy, the trend from 2017 to 2023 revealed a noteworthy regional average increase from 44.4% to 54.9% (Figure 2). In 2023, the active recall strategy was directed towards individuals reaching the age of 65, with a particular focus on those born in 1958.

### 3.2. Family Pediatricians for Childhood Vaccinations

**Figure 3:**
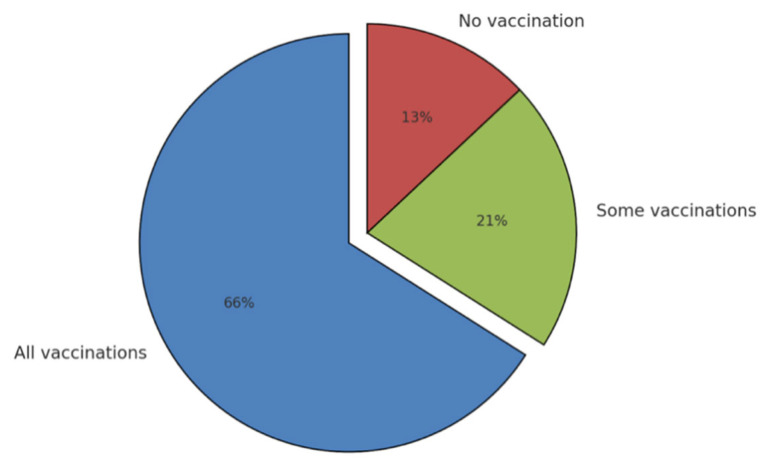
Vaccination activity of family pediatricians.

The Tuscany region (central Italy, 3.7 million inhabitants) was the first (and currently the only one) region to proactively engage family pediatricians in the administration of childhood vaccines. Through an agreement between the region and family pediatricians, formalized on 30 October 2017 [34], there has been relevant physician involvement (Figure 3). In the initial two-year period following the implementation of this novel delivery model (2017 and 2018), family pediatricians were responsible for administering 64% of all vaccinations to children within the 0–14 age class. The effectiveness of this model was demonstrated by its ability to achieve the coverage levels set by the PNPV for childhood vaccinations. Despite the success of this vaccination delivery method in Tuscany, it has not yet been adopted by other regions.

### 3.3. Pharmacies and Vaccines for Influenza and COVID-19

The systematic involvement of service pharmacies as vaccination sites was established by a 2021 national law approved by the Parliament during the COVID-19 mass vaccination campaign [35,36]. The approach has been implemented in various ways across Italian regions, with an observed upward trend (Table 1). Until the 2023–2024 winter season, the vaccines administered in pharmacies were limited to seasonal influenza and COVID-19 booster doses. In 2024, the proportion of individuals who received the SARS-CoV-2 vaccination at a pharmacy ranged from 0% (in regions lacking specific agreements with pharmacies) to 70.1% in Liguria. This latter region, along with Lombardy (55.5%), adopted this strategy extensively with the support of local agreements with pharmacists’ associations [37] (Table 2). Regarding influenza vaccination, the rates of administration by pharmacists were lower. The highest rates were observed in Liguria [31] (12%) and Lombardy [31,38] (16%). In 2024, some regional experiments began, which also included the administration of anti-pneumococcal, anti-HPV, and anti-zoster vaccines in pharmacies. The results of these experiments will have to be evaluated over time, considering the new organizational issues, communication with the population, the creation of synergies between pharmacists and local health authorities (departments of prevention), and sufficient vaccine supplies.

### 3.4. Schools for HPV Vaccination

**Figure 4:**
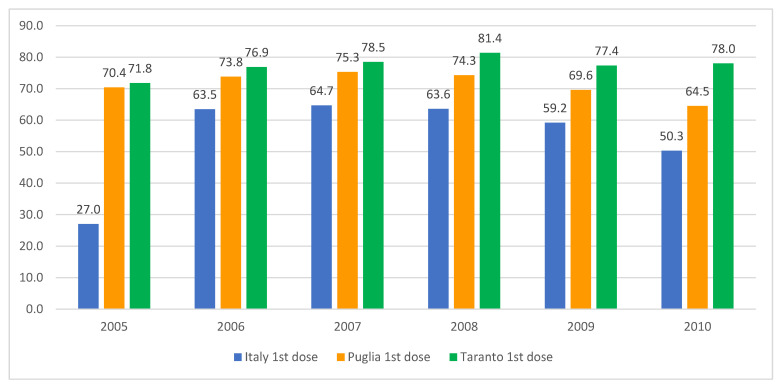
HPV vaccination coverage rates for females born between 2005 and 2010, detailing the uptake of the first and second doses in Italy, the Apulia region, and the city of Taranto. Sources: Ministry of Health and GIAVA.

**Figure 5:**
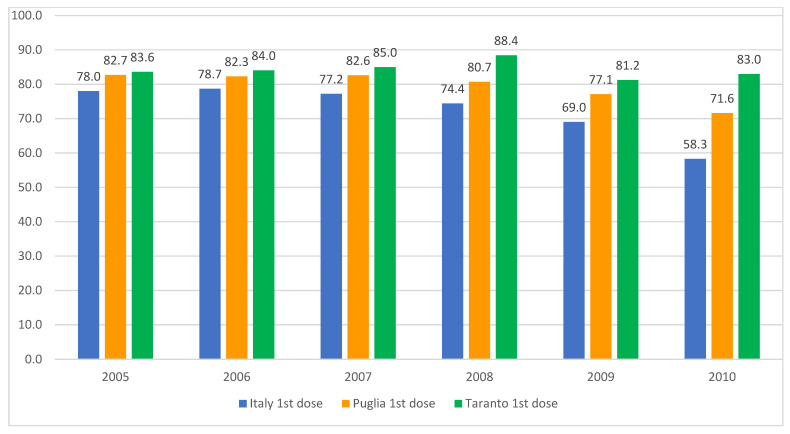
HPV vaccination coverage rates for males born between 2005 and 2010, detailing the uptake of the first and second doses in Italy, the Apulia region, and the city of Taranto. Sources: Ministry of Health and GIAVA.

Following the implementation of the 1978 health reform, the role of school doctors in Italian schools was dismissed, with their responsibilities assumed by family pediatricians and the prevention departments of local health authorities. Nevertheless, certain regions have endeavored to utilize schools as a conduit for the administration of the HPV vaccine to adolescents. The data from the Taranto Health Authority (southern Italy, population 0.6 million) demonstrate that the coverage rates for the initial dose of the HPV vaccine among females (Figure 4) and males (Figure 5) in the adolescent age group are markedly higher than both the national and regional averages. This underlines the potential efficacy of this approach, as detailed in an internal report from the local health authority of that province.

### 3.5. Hospital Vaccination Settings for Healthcare Workers and Vulnerable Individuals

All Italian hospitals are required to offer vaccinations to healthcare workers. However, prior to the global pandemic caused by the SARS-CoV-2 virus, it was uncommon for such institutions to establish dedicated vaccination centers for the general public and for patients in hospitals. The influenza vaccination coverage among healthcare workers remained low during the 2023/24 winter season, with a coverage rate below 40% at the national level, with only a few notable exceptions. A survey of 250 Italian hospitals conducted by the Association of Italian Medical Directors of Hospitals (ANMDO) revealed that 92.8% of hospitals offered vaccinations to healthcare workers, 76% also offered them to administrative staff, 57.6% to inpatients, and only 32.4% to outpatients [39]. A recent initiative, the Ospivax national project [40], aims to collaborate with hospitals that have commenced offering extended vaccination services in conjunction with the prevention departments of local health authorities. These vaccinations are provided directly to vulnerable patients within the hospital setting. While there are encouraging data on the administration of the COVID-19 vaccine in hospital hubs, systematic data on the administration of other vaccines in hospitals are not yet available.

## 4. Discussion

The Italian National Health Service, with significant regional levels of autonomy in providing healthcare services, is a noteworthy case study in the context of vaccination delivery. The five case studies analyzed in this paper illustrate the potential for regional autonomy to facilitate the development of innovative practices that are tailored to local needs, and which ultimately lead to an improvement in the level of immunization coverage. These good practices provide a model for overcoming some of the major obstacles faced by national immunization programs, including logistical difficulties, shortage of healthcare personnel, and vaccine hesitancy.

The decentralized nature of Italy’s healthcare system provides valuable parallels with other countries that employ regional autonomy to tailor public health strategies to local needs. Countries such as Germany and the United States offer insights into how decentralized frameworks can be leveraged to design effective vaccination programs. By examining these examples, it becomes evident that regional flexibility, guided by national recommendations, can enhance public health outcomes while addressing local challenges. Germany’s decentralized healthcare system mirrors Italy’s regional autonomy [41]. Federal states (*Länder*) implement vaccination policies based on national recommendations from the Standing Committee on Vaccination (STIKO) [42] and directives from the Federal Joint Committee [43], whereas the United States exemplifies regional autonomy in vaccination policies [44], with individual states determining mandatory vaccines for school enrollment [45,46] based on national recommendations from the Centers for Disease Control and Prevention (CDC) [47].

One of the pivotal findings derived from these case studies is the efficacy of targeted interventions in enhancing vaccination coverage for specific population groups. The vaccination services in the Veneto region, managed by the departments of prevention of local health authorities, have achieved significant results for the HPV vaccine, which remains relatively unknown in the population and underutilized. This success exemplifies how a focused, regional strategy, supported by administrative commitment and active recall systems, can markedly enhance vaccination coverage. This may be attributed to the region’s robust tradition of territorial services, which provides greater resources (such as doctors and healthcare assistants). This model could serve as a reference for other regions, particularly those experiencing difficulties in achieving adequate vaccination coverage among adults and the elderly.

Similarly, the involvement of family pediatricians in Tuscany’s childhood vaccination programs exemplifies the potential of leveraging existing healthcare networks to improve service delivery. By integrating family pediatricians into the vaccination system, Tuscany was able to exceed national targets for childhood immunizations, thereby demonstrating the pivotal role that locally based, physician-led initiatives can play in enhancing access to vaccines for young children. This approach remains neglected in other regions, indicating that there is considerable scope for its wider adoption to enhance national childhood vaccination outcomes.

The local health authority vaccination centers continue to serve as the primary framework for the administration of childhood and adolescent vaccinations. Nevertheless, it has become increasingly evident that alternative vaccination settings are indispensable for reaching underserved populations and improving accessibility, particularly by strengthening catch-up vaccination efforts [48,49,50]. Alternative settings, including pharmacies, hospital outpatient clinics, and educational institutions, have demonstrated efficacy in enhancing vaccination coverage, particularly among populations with limited access to healthcare services and those at elevated risk of vaccine-preventable illnesses. For example, northern Italy shares borders with France, Switzerland, Austria, and Slovenia, leading to frequent cross-border movement for work, tourism, and trade. The free movement within the Schengen Area necessitates robust vaccination coverage to prevent the transnational spread of diseases [51,52,53]. In these northern regions, alternative vaccination settings have proven effective in reaching mobile and underserved populations. These settings provide families with enhanced convenience, thereby reducing logistical barriers such as the necessity for them to return to their usual care providers, which is a recognized barrier to the completion of vaccination schedules [54,55,56]. On the other hand, southern Italy, particularly regions such as Sicily, Calabria, and Apulia, serves as a primary gateway for migrants arriving from Africa. This influx presents unique challenges and opportunities for vaccination efforts. Migrants may come from countries with different vaccination schedules or lower overall vaccination coverage, potentially increasing the risk of vaccine-preventable diseases re-emerging or spreading within Italy [57]. To minimize the risk, Italian health authorities have implemented targeted vaccination programs within reception centers and migrant communities [58,59,60]. These programs include comprehensive health assessments and the administration of vaccinations according to the national schedule, regardless of the migrants’ legal status [61,62]. Actually, Italian law guarantees access to essential healthcare services, including vaccinations, for all individuals present in the country, regardless of their immigration status. Partnerships with non-governmental organizations (NGOs), the Red Cross, and agencies such as the International Organization for Migration (IOM) facilitate vaccination efforts by providing resources, cultural mediation, and logistical support [63].

Furthermore, the COVID-19 pandemic not only demonstrated the vulnerability of relying solely on traditional healthcare environments but also underscored the necessity of diversifying vaccine delivery platforms. In Italy, the inclusion of pharmacies as vaccination providers has become a pivotal component of the national strategy, particularly during the COVID-19 vaccination campaign [56]. The use of non-healthcare facilities for vaccine administration, such as pharmacies and schools, requires careful central coordination by the region and local health authorities. Nevertheless, this approach demonstrates considerable potential, as highlighted by the data from Taranto for schools and from the Lombardy and Liguria regions concerning pharmacies. The success of these efforts points to the potential for expanding pharmacy-administered vaccinations to include a wider range of vaccines, such as those against pneumococcal disease, zoster, and HPV. This approach would result in an overall increase in vaccination rates, facilitating improved access for populations in rural or underserved areas (small islands and mountain locations). Pharmacies have a broader reach than other healthcare providers, and individuals are more likely to be familiar with and trust their local pharmacists [64,65]. Furthermore, pharmacies have the potential to offer opportunistic vaccinations to individuals seeking other health services [66,67]. The combined effect of these factors would assist Italy in achieving the objectives set out in its PNPV 2023–2025 [68]. Currently, pharmacists are authorized to provide vaccinations in 15 European countries, primarily against influenza and COVID-19. Furthermore, in more than half of these countries, pharmacists are also permitted to administer a broader range of vaccines, including those for pneumococcal disease, herpes zoster (shingles), cholera, diphtheria, tetanus, pertussis, meningococcal disease, tick-borne encephalitis, typhoid fever, hepatitis A and B, human papillomavirus (HPV), rabies, human rotavirus, and varicella [69].

For example, in Denmark, pharmacists began offering influenza vaccinations in 2014, with services supported by mandatory training and integration into a national vaccination database. COVID-19 vaccinations were added in 2021 under physician delegation. Similarly, France launched pharmacist vaccination services nationally in 2019 after a successful pilot, and by 2022, pharmacists administered 60% of COVID-19 vaccines. New legislation now allows French pharmacists to prescribe and administer most vaccines for individuals aged 11 and older. In Germany, pharmacist-led flu vaccination pilots started in 2020 and expanded to nationwide availability by 2022/2023, with pharmacists also joining the COVID-19 vaccination strategy after completing comprehensive training. In Ireland, pharmacy-based vaccination services began in 2011 and have since expanded to include flu, shingles, pneumococcal, and COVID-19 vaccines, with pharmacists playing a significant role in national immunization programs. These services have consistently demonstrated high patient satisfaction, streamlined record-keeping through digital systems, and improved coverage, particularly for populations previously underserved by traditional healthcare settings [70,71].

Finally, the presented examples illustrate that hospital vaccination centers can be established through a collaboration between local health authorities and specialists in hygiene and preventive medicine from university hospitals. This setting could be highly beneficial in reaching the targets set for vulnerable populations (the elderly and those with chronic conditions). Nevertheless, the number of such cases is currently limited within the country, and problems related to privacy can reduce the possibility of reaching vulnerable population groups.

### Limitations and Recommendations

This study identified several critical limitations that must be addressed to optimize vaccination delivery across Italy. A key challenge is the regional variability in the implementation of vaccination strategies, particularly regarding the involvement of alternative providers, such as pharmacies. While regions such as Liguria and Lombardy have successfully integrated pharmacies into vaccination campaigns, other regions have been slower to adopt this model, resulting in suboptimal access to vaccines. The absence of a uniform policy framework and discrepancies in regional agreements between pharmacies and health authorities undermine the broader effectiveness of this approach.

To further enhance vaccine delivery in alternative settings, several factors need to be prioritized. It is crucial to ensure the provision of adequate funding and reimbursement for the supply, storage, administration, and counselling of vaccines to guarantee the long-term sustainability of these settings. Pharmacies, schools, and other non-traditional vaccination venues require not only support for vaccine acquisition but also for the necessary infrastructure to safely store and administer vaccines. This includes investments in vaccine storage systems, the recruitment of a sufficient number of personnel, and the implementation of comprehensive training programs for said personnel.

A further key element is the training of staff in alternative settings. It is imperative that healthcare providers possess comprehensive knowledge of the most recent vaccination recommendations, including any contraindications, and have received training in how to address common concerns, particularly those related to vaccine hesitancy. The COVID-19 pandemic has demonstrated the necessity for resilient approaches to healthcare provision, emphasizing the importance of effective communication training to address public misconceptions about vaccines. Furthermore, vaccination initiatives in these settings should be closely aligned with primary care providers to guarantee the continuity of care and the maintenance of accurate vaccine records.

Another limitation concerning the fragmentation of vaccine administration is the lack of access to a national vaccination registry available to all vaccinators for digitally recording vaccinations. As a result, vaccinations must be communicated to the local health authority (ASL), which is then responsible for recording it in the regional portal, connected with the national register. A possible solution would be to establish a digital vaccination registry that is widely accessible, regardless of where the vaccine is administered. This would allow for the real-time recording of vaccinations, ensuring that data are immediately available to the local health authority and integrated into the regional and national vaccination systems. Such a system would streamline communication and reduce administrative delays, which can introduce biases in the calculation of vaccination coverage. This lag in data reporting can lead to inaccurate assessments of vaccine uptake, potentially affecting public health planning and the timely identification of gaps in coverage.

Finally, workforce shortages and logistical challenges, particularly in rural or underserved areas, represent barriers to achieving comprehensive vaccine coverage. In such areas, both traditional and alternative vaccination providers are often underutilized, underscoring the necessity for targeted interventions. Addressing these limitations is critical to meet the targets of the PNPV 2023–2025 and improve overall public health outcomes.

## 5. Conclusions

This study showed the potential of regionally tailored vaccination strategies in enhancing immunization coverage and addressing challenges within the Italian healthcare system. By analyzing five best practices, we showed how regional autonomy fosters innovation and adaptability in vaccine delivery. Key findings include the success of active recall strategies in Veneto, the integration of family pediatricians in Tuscany, and the pivotal role of pharmacies in Liguria and Lombardy in increasing access to vaccines. Additionally, school-based HPV vaccination programs in Taranto and hospital-centered initiatives for healthcare workers and vulnerable populations underline the importance of leveraging alternative settings for immunization. These examples illustrate that utilizing different vaccination providers allows local authorities to address logistical challenges, workforce shortages, and vaccine hesitancy, while still achieving national immunization targets. While regional differences must be carefully considered when implementing such strategies, the practices analyzed in this study provide a valuable reference for developing new delivery models. Integrating these findings into national frameworks, supported by consistent data collection and collaboration among stakeholders, will be crucial for optimizing vaccination strategies and ensuring equitable access to healthcare. Future research should further explore the scalability and long-term sustainability of these approaches to inform global vaccination policy.

## Figures and Tables

**Table 1:** SARS-CoV-2 vaccine doses administered in pharmacies and their relative contribution to the national vaccination campaign.

Year	Total Doses Administered (Millions)	Pharmacy Doses (Millions)	Pharmacy Contribution (% of Total Doses)
2021	111	1.4	1%
2022	33	2.6	8%
2023	2.7	0.62	23%
2024	0.27	0.08	30%

**Table 2:** Pharmacies’ contribution to the administration of SARS-CoV-2 vaccine doses by region (relative %).

Region	2021	2022	2023	2024
Piedmont	2.1	6.6	23.8	30.9
Aosta Valley	0.8	6	1.2	0
Lombardy	0.5	14	47.8	55.5
Prov. Auton. Bolzano	0.7	6.4	5.4	23.6
Prov. Auton. Trento	0	0	0	0
Veneto	0.9	2.9	11.5	19.5
Friuli Venezia Giulia	0	0.1	18	28.4
Liguria	8.8	21.2	58.9	70.1
Emilia Romagna	0.1	1	5.9	12.8
Tuscany	1	5.3	4.6	7.2
Umbria	0.9	9.2	11	12.1
Marche	2.8	10.3	31.4	51.3
Lazio	2.3	8.6	21.7	39.7
Abruzzo	2.3	9.5	18.2	25.8
Molise	0	0	0	0
Campania	1.4	7.8	20.9	24.2
Apulia	0.7	10	10.6	15.5
Basilicata	0	0	0	0
Calabria	1.4	6.9	11.6	18.9
Sicily	0.9	8.7	11.9	6.2
Sardinia	0	0	0	0
Total	1.3	7.9	23	30.3

## Data Availability

The data that support the findings of this study are not publicly available but can be provided upon reasonable request to Carlo Signorelli, subject to the provision of a justified rationale and approval for their use.
